# COVID-19 among undocumented migrants admitted to French intensive care units during the 2020–2021 period: a retrospective nationwide study

**DOI:** 10.1186/s13613-023-01197-8

**Published:** 2023-10-06

**Authors:** Sami Hraiech, Vanessa Pauly, Véronica Orleans, Pascal Auquier, Elie Azoulay, Antoine Roch, Laurent Boyer, Laurent Papazian

**Affiliations:** 1 Service de Médecine Intensive - Réanimation, AP-HM, Hôpital Nord, Marseille, France; 2 Faculté de medecine, Centre d’Etudes et de Recherches sur les Services de Santé et qualite de vie EA 3279, Aix-Marseille Université, 13005 Marseille, France; 3 Départment d’Informatique Médical, AP-HM, Marseille, France; 4 Centre Hospitalier de Bastia, 20600 Bastia, Corsica France; 5 Service de Médecine Intensive et Réanimation, Hôpital Saint-Louis, AP-HP, Paris, France

**Keywords:** Undocumented migrants, Refugees, Asylum seekers, COVID-19, Intensive care unit, Acute respiratory distress syndrome, Mechanical ventilation, Mortality

## Abstract

**Background:**

Before the Coronavirus Disease 2019 (COVID-19) pandemic in France, undocumented migrants had a higher risk than general population for being admitted to the intensive care unit (ICU) because of acute respiratory failure or severe infection. Specific data concerning the impact of COVID-19 on undocumented migrants in France are lacking. We aimed to analyze the mortality and respiratory severity of COVID-19 in this specific population.

We retrospectively included all undocumented adult migrants admitted in French ICUs from March 2020 through April 2021 using the French nationwide hospital information system (Programme de Médicalisation des Systèmes d’Information). We focused on admissions related to COVID-19. Undocumented migrants were compared to the general population, first in crude analysis, then after matching on age, severity and main comorbidities. The primary outcome was the ICU mortality from COVID-19. Secondary objectives were the incidence of acute respiratory distress syndrome (ARDS), the need for mechanical ventilation (MV), the duration of MV, ICU and hospital stay.

**Results:**

During the study period, the rate of ICU admission among patients hospitalized for COVID-19 was higher for undocumented migrants than for general population (463/1627 (28.5%) vs. 81 813/344 001 (23.8%); *p* < 0.001). Although ICU mortality was comparable after matching (14.3% for general population vs. 13.3% for undocumented migrants; *p* = 0.50), the incidence of ARDS was higher among undocumented migrants (odds ratio, confidence interval (OR (CI)) 1.25 (1.06–1.48); *p* = 0.01). Undocumented migrants needed more frequently invasive MV (OR (CI) 1.2 (1.01–1.42); *p* = 0.04 than general population. There were no differences between groups concerning duration of MV, ICU and hospital length of stay.

**Conclusion:**

During the first waves of COVID-19 in France, undocumented migrants had a mortality similar to the general population but a higher risk for ICU admission and for developing an ARDS. These results highlight the need for reinforcing prevention and improving primary healthcare access for people in irregular situation.

**Supplementary Information:**

The online version contains supplementary material available at 10.1186/s13613-023-01197-8.

## Background

The part of undocumented migrants (UM) admitted to French intensive care units (ICUs) has been constantly increasing during the last decade [1] despite the so-called “healthy migrants hypothesis” [2]. Harshness of the travel and living conditions as well as barriers to primary healthcare access at least partly explain the frailty of undocumented people, asylum seekers and refugees towards critical illness [3]. Before the Coronavirus Disease 2019 (COVID-19) pandemic, in France, undocumented migrants had a higher risk than general population for being admitted to the ICU because of acute respiratory failure or severe infection [1]. COVID-19 has severely impacted migrants’ health worldwide [4]. International reports suggest that ethnic minorities, and foreign born patients have a higher risk for COVID-19 infection [5] and hospitalization [6]. Variable rates of ICU mortality have been reported according to world regions [6–10]. The heterogeneity of migrants’ definitions used, confounding factors such as severity of the disease and the specificities of healthcare systems might explain these discrepancies. French registers argue for a higher mortality among foreign born patients [11] but specific data on undocumented migrants are lacking. We therefore decided to analyze the impact of COVID-19 during the first months of pandemic in France focusing specifically on critically ill undocumented migrants.

Our primary objective was to compare the ICU mortality from COVID-19 of undocumented migrants and general population. Secondary objectives were to compare the severity of the respiratory disease, as reflected by the rate of acute respiratory distress syndrome (ARDS), the need for invasive mechanical ventilation and the duration of MV, ICU and hospital stay.

## Material and methods

### Study population

All the adult patients admitted to French hospitals during the study period were identified using the French nationwide hospital information system (Programme de Médicalisation des Systèmes d’Information), which collects administrative and medical information for all hospital stays regardless of hospital funding. Diagnosis-related groups are recorded according to ICD-10 criteria and life-supporting treatments according to the French classification of medical interventions (Classification Commune des Actes Médicaux). The study periods corresponded to the first months of COVID-19 pandemic in France, usually called “waves”:

- 1st wave: from March 1st to May 31st 2020
- 2nd wave: from September 1st to December 31st 2020
- 3rd wave: from January 1st to April 30th 2021.

Only hospital stays in conventional ward (surgical, medical and obstetrics) or intensive care unit were considered. Patients admitted for ambulatory care were excluded. In case of inter-hospital transfers (sequential and continuous hospitalizations), the 2 stays were considered as a single one with a duration equal to the sum of the hospitalization durations in the different hospitals. Consequently, a patient transferred from one ICU to another in a different hospital was considered as a single hospitalization resuming the information gathered during the two stays.

### Case ascertainment

Undocumented migrants and general population were identified into the study population using the French health insurance system. The agency within the statutory health insurance system that funds inpatient care is recorded as usual health insurance (available to all French citizens and non-French people who have residence permits). For undocumented residents who entered France less than or at least 3 months earlier, the device is called, respectively, “urgent care” or “state medical aid”. State medical aid ensures a free takeover of all type of healthcare. Urgent care ensures a free takeover for acute healthcare only. Patients benefiting from both device can have free COVID-19 testing and treatments.

All data are anonymized and are accessible to accredited researchers committed to following database-use guidelines [12].

All patients admitted to the hospital during the study period were identified, and their health insurance status was recorded. Patients covered by the usual health insurance system formed the general population. Patients covered by either of the systems for undocumented residents (urgent care or state medical care) formed the undocumented migrants group. Patients not covered by any of the French statutory health insurance systems were excluded.

Inside this study population, we focused on COVID-19 related hospitalizations. Patients hospitalized for COVID-19 were identified using the following ICD-10 codes: U07.10; U07.11; U07.14; U07.15. We then compared the characteristics and outcomes of patients admitted to the ICU for COVID-19 according to the undocumented migrants status (vs. general population).

### Data collection and outcomes

For each patient admitted to the ICU for COVID-19, we used standardized forms with extraction from nationwide hospital information system to collect the following data; admissions within the past year, admission from home or another hospital department, geographical location of the hospital, type of hospital (public university-affiliated or non-university-affiliated hospital, public community hospital, private for-profit or not-for-profit hospital, national cancer center, or military hospital), and some specific comorbidities from the modified Charlson Comorbidity Index [13] and Elixhauser score [14] also computed from the ICD-10 codes recorded as primary or secondary diagnoses during the last 3 months of life.

For patients in the ICU, the evolution towards ARDS was noted. Life-supporting interventions such as high-flow nasal oxygen (HFNO), non-invasive ventilation (NIV), invasive mechanical ventilation (MV), septic shock, renal replacement therapy (RRT), extracorporeal membrane oxygenation, extracorporeal CO2 removal were derived from coding acts (Additional file 1).

The following outcomes were also computed: ICU and hospital mortality, duration of mechanical ventilation, duration of ICU and hospital stay.

The primary outcome was the ICU mortality from COVID-19 among undocumented migrants and general population. Secondary objectives were the severity of the respiratory disease, as reflected by the rate of acute respiratory distress syndrome (ARDS) and the need for invasive mechanical ventilation in ICU, duration of MV, ICU and hospital lengths of stay.

### Comparisons of undocumented migrants and general population

We first performed a crude analysis on the whole population admitted in ICU comparing undocumented migrants to the general population. The data were described as mean ± SD or median [interquartile range] after assessment of distribution normality for quantitative variables and frequencies and percentages for qualitative ones. We used Chi-square test or Fisher exact test to compare both populations among qualitative variables and Student’s *t* test with Welch’s correction if applicable for quantitative ones. We also computed standardized differences (SDs) which is an effect-size method to identify between-group differences that, unlike P values, are not influenced by sample size. Values greater than 0.10 are considered as clinically significant [15].

### Analysis of discrepancies of ICU admission between groups (undocumented migrants vs general population)

To study ICU admission rates, we performed a matched-control analysis among patients admitted in ICU. Migrants were matched to general population patients (exact matching of 1 migrant for up to 5 non migrant) on the following criteria: age (± 5 years), sex, Simplified Acute Physiology Score version II (SAPSII) without the age points (± 10), type of hospital, geographical area on the French territory (department), COVID-19 “wave”, COPD, comorbidities (at least 3 of the following: diabetes, obesity, chronic kidney disease, chronic liver disease, malignancy, alcohol abuse, smoker status).

Weights were applied to normalize the distribution of patients due to unequal matching numbers across matched migrants. Migrant people with no matched-control eligible were removed from the analysis. The remaining data formed the matched cohort, within which we compared the distribution of covariates between migrants and general population, by computing the SDs and taking into account weights.

To assess associations between migrant status and outcomes, we fit Generalized Estimating Equation (GEE) models for repeated measures in the matched cohort, using logistic models for binary outcomes and linear log-normal-distributed models for quantitative outcomes. Associations were evaluated by computing odds ratios (ORs) for binary outcomes and back-transformed beta coefficients for quantitative outcomes, both with their 95% confidence intervals (95% CIs).

The statistical analysis was performed using PROC GLIMMIX in SAS^®^ (Cary, NC). All tests were two-sided, and *P* values smaller than 0.05 were considered to indicate statistically significant differences.

## Results

**Fig. 1:**
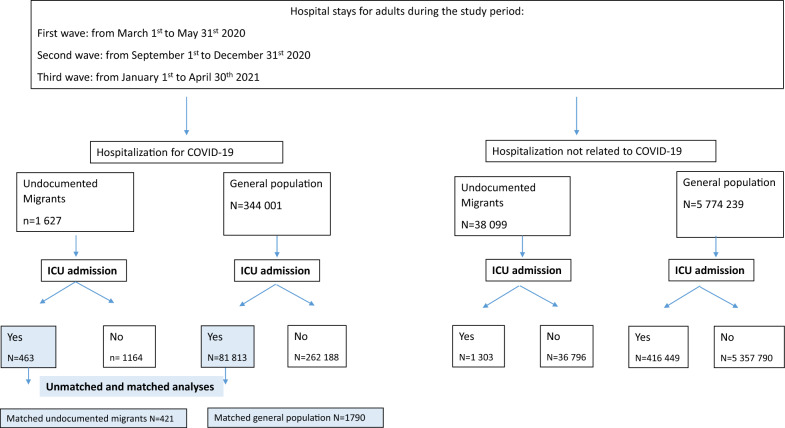
Study flowchart. *ICU* intensive care unit

Figure 1 represents the study flowchart.

During the study period, 4.1% (1.627/39.726) of the hospitalizations among undocumented migrants were due to COVID-19 as compared to 5.6% (344.001/6.118.240) in the general population (*p* < 0.001). The rate of ICU admission among COVID-19 patients was higher for undocumented migrants than for general population (463/1627 (28.5%) vs. 81.813/344.001 (23.8%); *p* < 0.001). Conversely, for patients not hospitalized for COVID-19, the rate of ICU admission was lower for undocumented migrants than for general population (1.303/38.099 (3.4%) vs. 416.449/5.774.239 (7.2%); *p* < 0.001).

Table 1 describes the characteristics of undocumented migrants and general population admitted to the ICU for COVID-19, in the unmatched and matched analyses. Hypertension, obesity and *diabetes mellitus* were the most frequent comorbidities in both migrants and general population, although migrants being younger had less comorbidities. Migrants were more frequently admitted directly to the ICU and exhibited a shorter delay from hospital to ICU admission. These results were found both in the crude and matched analyses.

**Table 1:** Patient’s characteristics at ICU admission in the unmatched and matched cohorts

	Unmatched analysis					Matched analysis				
*N* (%) or mean ± SD	General population		Migrants		SMD	General Population		Migrants		SMD
	*N* = 81 813		*N* = 463			*N* = 1 790		*N* = 421		
Sex (*n*, % or weighted^a^ %)					0.0471					0
Males	53.268	65.1	291	62.9		1.170	63.7	268	63.4	
Females	28.545	34.9	172	37.1		620	36.3	153	36.3	
Age, years (mean ± SD)	66 ± 14		54 ± 15		0.8129	55 ± 7		55 ± 15		0.0278
SAPS II (without age) (mean ± SD)	22 ± 16		23 ± 15		0.0146	22 ± 14		21 ± 6		0.0402
Comorbidities (*n*, % or weighted^a^ %)										
Chronic heart failure	15.505	19	51	11	0.2238	195	10.9	46	10.9	0.0004
COPD	13.193	16.1	29	6.3	0.3167	54	4.5	19	4.51	0.0222
Chronic kidney disease	10.229	12.5	38	8.2	0.1413	126	7.9	34	8.1	0.0073
Liver disease	4.641	5.6	24	5.2	0.020	94	5.5	21	4.9	0.021
Cancer	8.423	10.3	31	6.7	0.1294	114	6.7	28	6.7	0.001
Diabetes mellitus	23.382	28.6	106	22.3	0.1303	445	24.5	98	23.3	0.028
Obesity	25.340	31	124	26.8	0.0926	569	32	107	25.4	0.1451
Hypertension	40.548	49.6	179	38.7	0.2209	654	36.2	166	39.4	0.0677
Smoker	6.370	7.8	19	4.1	0.1562	104	6.2	18	4.3	0.0856
Alcohol abuse	3.909	4.8	16	3.5	0.0666	51	2.9	15	3.6	0.0376
Hospital category (*n*, % or weighted^a^ %)					0.7207					0
Public, non-university	38.989	47.7	181	39.1	0.1734	639	37.1	156	37.1	
Public, university	26.915	32.9	154	33.3	0.0077	621	33.7	142	33.7	
Private	15909	19.5	128	27.7	0.1942	530	29.2	123	29.2	
Admission conditions (*n*, % or weighted^a^ %)										0
First wave	22.356	27.3	178	38.4	0.2384	687	38.7	163	38.7	
Second wave	21.739	26.6	99	21.4	0.1218	324	19.5	82	19.5	
Third wave	37.718	46.1	186	40.2	0.1199	779	41.8	176	41.8	
Direct admission to the ICU, (*n*, % or weighted^a^ %)	33.302	40.7	246	53.1	0.251	792	43.14	224	53.2	0.2024
Time from hospital to ICU admission, days (mean ± SD)	2.2 ± 4.7		1.6 ± 3.6		0.1421	1.9 ± 2.1		1.6 ± 3.7		0.1089

*ARDS* acute respiratory distress syndrome, *ECMO* extracorporeal membrane oxygenation, *ECCO2R* extracorporeal CO_2_ removal, *ICU* intensive care unit, *NIV* non-invasive mechanical ventilation MV, mechanical ventilation, *RRT* renal replacement therapy, *SAPS II* Simplified Acute Physiology Score version II, *SMD* standardized mean difference

^a^In the matched analysis

### Outcomes and life support during the ICU stay

Patient’s outcomes are detailed in Table 2. Crude ICU mortality was higher among general population (19.263 (23.6%) vs. 63 (13.6%); SMD = 0.2577). After matching, there was no difference between groups concerning ICU mortality (258 (14.3%) for general population vs. 56 (13.3%) for undocumented migrants; *p* = 0.50). The incidence of ARDS was higher among undocumented migrants (odds ratio, confidence interval (OR (CI)) 1.25 (1.06–1.48); *p* = 0.01). Undocumented migrants needed more frequently invasive MV (OR (CI) 1.2 (1.01–1.42); *p* = 0.04 than general population whereas general population had a more frequent recourse to NIV (29.6% vs. 22.6%; *p* < 0.001). There were no differences between groups concerning duration of MV, ICU and hospital length of stay.

**Table 2:** Patients’ respiratory support and outcomes in the unmatched and matched cohorts

	Unmatched analysis			Matched analysis				
	General population (*N* = 81.813)	Migrants (*n* = 463)	SMD	General population (*n* = 1.790)	Migrants (*n* = 421)	SMD	*p*	Odds ratio or beta parameter (CI 95%)
Respiratory support (*n*, % or weighted^a^ %)								
ARDS	32.609 (39.9)	211 (45.6)	0.1157	734 (41.6)	198 (47)	0.1102	0.01	1.25 (1.06–1.48)
High flow oxygen	31.019 (37.9)	160 (34.6)	0.0699	625 (35.1)	150 (35.6)	0.0115	0.46	0.99 (0.86–1.09)
NIV	25.290 (30.9)	105 (22.7)	0.1867	521 (29.6)	95 (22.6)	0.1608	0.0002	0.69 (0.57–0.84)
Invasive MV	31.669 (38.7)	187 (40.4)	0.0344	624 (35.7)	168 (39.9)	0.0866	0.04	1.20 (1.01–1.42)
ECMO/ECCO_2_R	1.503 (1.8)	11 (2.4)	0.0375	43 (2.7)	11 (2.6)	0.0078	0.85	0.95 (0.57–1.6)
Outcomes [median [IQR] or *n* (%)]								
Invasive MV, days	14 [7–28]	15 [8–29]	0.0331	14 [7–27]	15 [8–29]	0.0097	0.47	1.06 (0.90–1.22)
ICU stay, days	8 [4–18]	8 [4–16]	0.0414	8 [4–16]	8[4–16]	0.0169	0.42	0.97 (0.88–1.04)
Hospital stay, days	16 [9–29]	14 [8–27]	0.0013	14 [9–25]	14 [8–26]	0.0875	0.57	0.98 (0.91–1.05)
ICU mortality	19.263 (23.6)	63 (13.6)	0.2577	258 (14.3)	56 (13.3)	0.029	0.50	0.919 (0.72–1.17)
Hospital mortality	22.088 (27)	68 (14.7)	0.3066	278 (15.7)	61 (14.5)	0.035	0.42	0.91 (0.72–1.15)

*ARDS* acute respiratory distress syndrome, *CI* confidence interval, *ECMO* extracorporeal membrane oxygenation, *ECCO2R* extracorporeal CO2 removal, ICU intensive care unit, *IQR* interquartile range, *NIV* non-invasive mechanical ventilation, *MV* mechanical ventilation, *SMD* standardized mean difference

^a^In the matched analysis

The incidence of septic shock and acute kidney failure with the need for renal replacement therapy were comparable in the general population and in migrants (458 (27.2%) vs. 121 (28.7%) patients; SMD = 0.0354 and 128 (7.9%) vs.33 (7.8%) patients; SMD = 0.0003).

## Discussion

We describe here the first series specifically focusing on undocumented migrants’ critical illness during the 1st waves of COVID-19 in France. Our results show that undocumented migrants had a mortality similar to the general population but were more frequently admitted to the ICU and developed more ARDS.

Our primary objective was to determine if undocumented migrants exhibited an excess of mortality from COVID-19 as compared to general population. This had been suggested by a previous French report [11] with a specific burden within the working-age range. However, these results were limited to the first wave of pandemic and to immigrants born outside Europe without any adjustment at the individual level. We did not confirm this difference of mortality. In a precedent nationwide study focusing on non COVID-19 French ICU patients [1], we already described that, despite migrants required more frequently vasopressors, mechanical ventilation and renal replacement therapy, they had similar mortality as compared to general population. It is plausible that, although more impacted by COVID-19, undocumented migrants are initially healthier than average which confers them a protective effect towards mortality. From an international point-of-view, conflicting data exist concerning the impact of COVID-19 on migrants’ mortality. The WHO European Region systematic review reported an association between mortality and the belonging to migrants and ethnic minorities group [4]. However, most data concerned the UK with large differences between ethnic groups. Reports from Northern Europe [9] and Canada did not confirm such results [16]. This geographical heterogeneity suggests a significant role of healthcare systems. Indeed, large discrepancies exist concerning the level of protection ensured for undocumented migrants in Western countries [17].

We also aimed to compare the severity of the respiratory disease, as reflected by the rate of ARDS and the need for invasive MV. We found that, even after adjustment, undocumented migrants had a higher incidence of ARDS, which was described in only one Koweitian series before ours [7]. A possible explanation for this result is that undocumented migrants were admitted to the hospital at a later stage of the disease with a more severe respiratory presentation and consequently exhibited higher rates of ICU admission, invasive MV and ARDS. In France, it was shown that migrants were more exposed to COVID-19-related factors [18], especially when housed in camps, because of promiscuity. Despite being younger and with fewer comorbidities, they may have experienced difficulties to healthcare access resulting in delayed treatments, as described for other diseases [19]. Language barrier, poor knowledge of healthcare facilities and social rights might explain that undocumented migrants have waited longer before seeking medical aid, perhaps fearing the consequences (particularly to be reported to immigration services and deported) [17]. Fabiani et al. [6] found that non-Italian COVID-19 cases were diagnosed at a later stage and were more likely to be hospitalized with differences being more pronounced in patients coming from countries with lower human development index. In our cohort, higher rates of direct admission to the ICU and shorter delay from hospital to ICU are indirect arguments for a more severe illness at hospital arrival. The higher recourse to invasive MV for undocumented migrants could argue for this hypothesis but this result has to be interpreted with caution considering the variability of intubation criteria among COVID-19 patients according to the period, centers and availability of resources [20].

In our cohort, undocumented migrants had higher rates of ICU admission. This was described in other European Union countries [4, 6] with up to fourfold increased risk of ICU admittance after adjusting for age in non-native people [21]. In a Canadian series, Passos-Castilho et al. [16] found similar results with discrepancies according to the country of birth. In a large WHO European Region systematic review, 15 studies out of 16 reported that migrants and ethnic minorities had a higher risk of hospital admission for COVID-19 compared to native/White ethnicity [4]. However, in these studies, undocumented migrants were not analyzed separately but together with non-native patients and ethnic minorities, regardless their legal status.

The main limitation of our study is its retrospective design. Furthermore, differences in region of birth, educational level, economic status, reasons for leaving the home country make the undocumented migrants population a very heterogeneous one, calling for caution when extrapolating results. Due to the study design, we could not collect some data of importance such as ICU non-admission decisions, especially at the beginning of pandemic when the strain on the healthcare system was particularly high, or specific SARS-CoV-2 treatments. Lastly, we did not report vaccination status. A reduced access to COVID-19 vaccines has been shown among migrants [22]. However, in our cohort, vaccination might have concerned only a few patients from the 3rd wave as it was generalized to all adults in France from May 2021.

## Conclusion

During the first waves of COVID-19 in France, undocumented migrants had a mortality similar to the general population but a higher risk for ICU admission and for developing an ARDS. These results highlight the need for reinforcing prevention and improving primary healthcare access for people in irregular situation.

### Supplementary Information

**Additional file 1.** French Classification of Medical Acts (CCAM: Classification Commune des Actes Médicaux) for ICU supportive therapies.

## Data Availability

The datasets used and/or analyzed during the current study are available from the corresponding author on reasonable request.

## Abbreviations

ARDS: Acute respiratory distress syndrome
ECMO: Extracorporeal membrane oxygenation
ECCO2R: Extracorporeal CO2 removal
ICU: Intensive care unit
NIV: Non-invasive mechanical ventilation
MV: Mechanical ventilation
RRT: Renal replacement therapy
SAPS II: Simplified Acute Physiology Score Version II
SMD: Standardized mean difference

## References

[1] Hraiech S, Pauly V, Orleans V, Auquier P, Boyer L, Papazian L (2022). Undocumented migrants in French intensive care units in 2011–2018: retrospective nationwide study. Intensive Care Med.

[2] Aldridge RW, Nellums LB, Bartlett S, Barr AL, Patel P, Burns R (2018). Global patterns of mortality in international migrants: a systematic review and meta-analysis. Lancet.

[3] Hraiech S, Papazian L, Azoulay E (2021). Migrants in the intensive care unit: time to show we care. Intensive Care Med.

[4] Mazzalai E, Giannini D, Tosti ME, D’Angelo F, Declich S, Jaljaa A (2023). Risk of Covid-19 severe outcomes and mortality in migrants and ethnic minorities compared to the general population in the European who region: a systematic review. J Int Migr Integr.

[5] Hayward SE, Deal A, Cheng C, Crawshaw A, Orcutt M, Vandrevala TF (2021). Clinical outcomes and risk factors for COVID-19 among migrant populations in high-income countries: a systematic review. J Migr Health.

[6] Fabiani M, Mateo-Urdiales A, Andrianou X, Bella A, Del Manso M, Bellino S (2021). Epidemiological characteristics of COVID-19 cases in non-Italian nationals notified to the Italian surveillance system. Eur J Public Health.

[7] Hamadah H, Alahmad B, Behbehani M, Al-Youha S, Almazeedi S, Al-Haddad M (2020). COVID-19 clinical outcomes and nationality: results from a Nationwide registry in Kuwait. BMC Public Health.

[8] Drefahl S, Wallace M, Mussino E, Aradhya S, Kolk M, Brandén M (2020). A population-based cohort study of socio-demographic risk factors for COVID-19 deaths in Sweden. Nat Commun.

[9] Holmberg V, Salmi H, Kattainen S, Ollgren J, Kantele A, Pynnönen J (2022). Association between first language and SARS-CoV-2 infection rates, hospitalization, intensive care admissions and death in Finland: a population-based observational cohort study. Clin Microbiol Infect.

[10] Patel A, Abdulaal A, Ariyanayagam D, Killington K, Denny SJ, Mughal N (2020). Investigating the association between ethnicity and health outcomes in SARS-CoV-2 in a London secondary care population. PLoS ONE.

[11] Khlat M, Ghosn W, Guillot M, Vandentorren S (2022). Impact of the COVID-19 crisis on the mortality profiles of the foreign-born in France during the first pandemic wave. Soc Sci Med.

[12] Homologation d’une méthodologie de référence relative aux traitements de données nécessitant l’accès par les établissements de santé et des fédérations aux données du PMSI et mises à disposition sur la plateforme sécurisée de l’ATIH. Disponible sur: https://www.legifrance.gouv.fr/affichTexte.do?cidTexte=JORFTEXT000037187535&categorieLien=id

[13] Charlson ME, Pompei P, Ales KL, MacKenzie CR (1987). A new method of classifying prognostic comorbidity in longitudinal studies: development and validation. J Chronic Dis.

[14] Elixhauser A, Steiner C, Harris DR, Coffey RM (1998). Comorbidity measures for use with administrative data. Med Care.

[15] Austin PC (2009). Using the standardized difference to compare the prevalence of a binary variable between two groups in observational research. Commun Stat Simul Comput.

[16] Passos-Castilho AM, Labbé AC, Barkati S, Luong ML, Dagher O, Maynard N (2022). Outcomes of hospitalized COVID-19 patients in Canada: impact of ethnicity, migration status and country of birth. J Travel Med.

[17] World Health Organization (Regional Office for Europe). Report on the health of refugees and migrants in the WHO European Region. 2018.

[18] Gosselin A, Warszawski J, Bajos N (2022). Higher risk, higher protection: COVID-19 risk among immigrants in France-results from the population-based EpiCov survey. Eur J Public Health.

[19] Winters M, Rechel B, de Jong L, Pavlova M (2018). A systematic review on the use of healthcare services by undocumented migrants in Europe. BMC Health Serv Res.

[20] Riera J, Barbeta E, Tormos A, Mellado-Artigas R, Ceccato A, Motos A (2023). Effects of intubation timing in patients with COVID-19 throughout the four waves of the pandemic: a matched analysis. Eur Respir J.

[21] Farrell RJ, O’Regan R, O’Neill E, Bowens G, Maclellan A, Gileece A (2021). Sociodemographic variables as predictors of adverse outcome in SARS-CoV-2 infection: an Irish hospital experience. Ir J Med Sci.

[22] Abba-Aji M, Stuckler D, Galea S, McKee M (2022). Ethnic/racial minorities’ and migrants’ access to COVID-19 vaccines: a systematic review of barriers and facilitators. J Migr Health.

