# Supplementary material for: COVID-19 among undocumented migrants admitted to French intensive care units during the 2020–2021 period: a retrospective nationwide study
Source: Ann Intensive Care. 2023 Oct 6;13:99. doi: 10.1186/s13613-023-01197-8 (PMC10558416; doi:10.1186/s13613-023-01197-8)
Supplement: Supplementary file 1 — Additional file 1. French Classification of Medical Acts (CCAM: Classification Commune des Actes Médicaux) for ICU supportive therapies. [file 13613_2023_1197_MOESM1_ESM.docx]

Appendix 1

**French Classification of Medical Acts (CCAM: Classification Commune des Actes Médicaux) for ICU supportive therapies.**

| **ICU supportive therapy** | **Classification according to French “CCAM”** |
| --- | --- |
| Catecholamines | EQLF003 |
| ExtraCorporeal CO_2_ Removal | GLJF010 |
| ExtraCorporeal Membrane Oxygenation | QGA001, EQQP004, EQLA001, EQLA002 |
| High flow nasal cannula | GLLD003 |
| Invasive Mechanical Ventilation | GLLD004, GLLD006, GLLD008, GLLD013, GLLD015 |
| Non-invasive Mechanical Ventilation | GLLD012, GLLD019 |
| Renal replacement therapy | JVJF002, 003, 004, 005, 008 |
